# Migrant status and risk of compulsory admission at first diagnosis of psychotic disorder: a population-based cohort study in Sweden

**DOI:** 10.1017/S0033291720002068

**Published:** 2022-01

**Authors:** J. Terhune, J. Dykxhoorn, E. Mackay, A.-C. Hollander, J. B. Kirkbride, C. Dalman

**Affiliations:** 1 PsyLife Group, Division of Psychiatry, UCL, London, W1T 7NF, UK; 2 CORE Group, Division of Psychology and Language Science, UCL, London, WC1E 7HB, UK; 3 EPICSS, Department of Global Public Health, Karolinska Institutet, Solnavägen 1E, SE-171 77 Stockholm, UK

**Keywords:** Compulsory admission, involuntary commitment, migration, neighbourhood, pathways to care, psychiatric epidemiology, psychosis, psychotic disorder, social determinants of health

## Abstract

**Background:**

Minority ethnic and migrant groups face an elevated risk of compulsory admission for mental illness. There are overlapping cultural, socio-demographic, and structural explanations for this risk that require further investigation.

**Methods:**

By linking Swedish national register data, we established a cohort of persons first diagnosed with a psychotic disorder between 2001 and 2016. We used multilevel mixed-effects logistic modelling to investigate variation in compulsory admission at first diagnosis of psychosis across migrant and Swedish-born groups with individual and neighbourhood-level covariates.

**Results:**

Our cohort included 12 000 individuals, with 1298 (10.8%) admitted compulsorily. In an unadjusted model, being a migrant [odds ratio (OR) 1.48; 95% confidence interval (CI) 1.26–1.73] or child of a migrant (OR 1.27; 95% CI 1.10–1.47) increased risk of compulsory admission. However after multivariable modelling, region-of-origin provided a better fit to the data than migrant status; excess risk of compulsory admission was elevated for individuals from sub-Saharan African (OR 1.94; 95% CI 1.51–2.49), Middle Eastern and North African (OR 1.46; 95% CI 1.17–1.81), non-Nordic European (OR 1.27; 95% CI 1.01–1.61), and mixed Swedish-Nordic backgrounds (OR 1.33; 95% CI 1.03–1.72). Risk of compulsory admission was greater in more densely populated neighbourhoods [OR per standard deviation (s.d.) increase in the exposure: 1.12, 95% CI 1.06–1.18], an effect that appeared to be driven by own-region migrant density (OR per s.d. increase in exposure: 1.12; 95% CI 1.02–1.24).

**Conclusions:**

Inequalities in the risk of compulsory admission by migrant status, region-of-origin, urban living and own-region migrant density highlight discernible factors which raise barriers to equitable care and provide potential targets for intervention.

## Introduction

There is strong evidence that risk of compulsory psychiatric care is greater for certain ethnic minority groups than majority white populations (Anderson, Flora, Archie, Morgan, & Mckenzie, 2014; Barnett et al., 2019; Weich et al., 2017). For example, in a recent international systematic review, Barnett et al. (2019) reported that compulsory inpatient admission was over twice as common for black compared with white populations, and between 1.33 and 2 times as common for people from Asian backgrounds. Migrants were 1.5 times more likely than non-migrants to experience compulsory detention for psychiatric care. This issue is particularly pervasive with respect to psychotic disorders, where several migrants and minority ethnic groups across international contexts face elevated incidence (Selten, Van Der Ven, & Termorshuizen, 2020), including in Sweden (Dykxhoorn et al., 2018), and increased risk of compulsory admission (Fassaert et al., 2016; Halvorsrud, Nazroo, Otis, Brown Hajdukova, & Bhui, 2018; Mulder, Koopmans, & Selten, 2006; Rodrigues et al., 2020; Rotenberg, Tuck, Ptashny, & McKenzie, 2017). Thus, people from many migrant and minority ethnic backgrounds may face the compound effects of inequalities in both risk of psychotic disorder and pathways to care. To date, however, most research on compulsory admission for psychotic disorders and ethnicity has been conducted in the UK (Halvorsrud et al., 2018), with fewer studies elsewhere, including the Netherlands (Fassaert et al., 2016; Mulder et al., 2006; Selten & Sijben, 1994) and Canada (Rodrigues et al., 2020; Rotenberg et al., 2017). In the UK, people from black ethnic backgrounds are between 2 and 5 times more likely to be compulsorily admitted than their white counterparts at first admission for psychosis (Cole, Leavey, King, Johnson-Sabine, & Hoar, 1995; Davies, Thornicroft, Leese, Higgingbotham, & Phelan, 1996; Mann et al., 2014; Morgan et al., 2005; Singh et al., 2015). Similar patterns have been observed in two studies in the Netherlands (Fassaert et al., 2016; Mulder et al., 2006), though not a third (Selten & Sijben, 1994). In Canada, Rodrigues et al. (2020) found African and Caribbean migrant groups to be at elevated risk even when compared with white European migrants, and Rotenberg et al. (2017) demonstrated risk of compulsory admission to be highest amongst people of East and South Asian origin.

Various hypotheses have been proposed to account for ethnic inequalities in compulsory admission for psychiatric care. These include the potential role of institutional racism (Keating, Robertson, McCulloch, & Francis, 2002; McKenzie & Bhui, 2007; Singh & Burns, 2006), attitudes towards mental health services (Sharpley, Hutchinson, McKenzie, & Murray, 2001) or greater social disadvantage (Burnett et al., 1999; Morgan et al., 2005; Rotenberg et al., 2017), which may all contribute to delays or barriers in obtaining timely, voluntary access to appropriate mental healthcare. These factors may contribute to evidence of differential pathways to care arising from greater police or judiciary involvement (Anderson et al., 2014; Rotenberg et al., 2017; Sharpley et al., 2001; Thomas, Stone, Osborn, Thomas, & Fisher, 1993) and greater subsequent risk of compulsory detention. Few studies have considered the role the broader social environment plays on compulsory admission for psychosis. Two notable exceptions include recent work by Rotenberg et al. (2017) in Canada, who found that compulsory admission for psychosis was higher in more residentially unstable communities (indexed by markers of social isolation, housing tenure and population turnover), and findings from Weich et al. (2017) in a nationwide study of all psychiatric admissions in England, who found that greater neighbourhood deprivation and ethnic density were associated with increased risk of compulsory admission.

Very little is currently known about whether migrant status (vis-à-vis ethnic minority status) is associated with greater risk of compulsory admission for psychosis. One study of any psychiatric disorder (not limited to psychosis) in Italy found no differences in compulsory admission for migrants (Tarsitani et al., 2013), but longitudinal evidence in large, nationwide samples is missing. Barnett et al. (2019) have emphasised the need to prioritise evidence from longitudinal studies, which have the potential to strengthen causal understanding as to whether cultural or structural (including sociodemographic) factors shape the risk of compulsory admission in migrant and minority ethnic groups.

Given the lack of such evidence with respect to first-episode psychosis, particularly in large samples, or from settings outside of the UK and Canada, we used longitudinal, nationwide data from Sweden to investigate whether (1) migrants and children of migrants were at greater risk of compulsory admission at first diagnosis of psychotic disorder than the Swedish-born population, and (2) if this was attributable to cultural or structural factors, including region-of-origin and own-region migrant density, or family income, neighbourhood population density, and deprivation, respectively. As in other countries, Sweden has a legal mechanism for compulsory psychiatric care via three health and social care acts, of which the Compulsory Psychiatric Care Act is most commonly enacted (Reitan, 2016). This allows registered doctors to issue a care certificate and detain individuals for up to 24 h for observation, on the grounds of serious mental health problems. An administrative court can then order compulsory psychiatric care for up to 4 weeks before review (Psykiatri Sydväst, 2020). Sweden does not record data on ethnicity, so throughout, we focus on differences in compulsory admission for migrants and their children compared with people born in Sweden to two Swedish-born parents (henceforth, the Swedish-born). We hypothesised that migrants and children of migrants would be more likely to be admitted compulsorily and that this risk would vary by region-of-origin, with highest risks in those from the Middle East and North Africa as well as sub-Saharan Africa, followed by Asian migrants, consistent with previous findings (Barnett et al., 2019; Rotenberg et al., 2017). We also hypothesised that higher own-region migrant density would be associated with a higher risk of compulsory admission in migrants and children of migrants and that these findings would persist after controlling for structural confounders including socioeconomic differences between these groups.

## Methods

### Study design and population

We extracted nationwide data from Psychiatry Sweden, an anonymized database of linked national registers. From the Register of the Total Population (RTP), we identified all individuals born between 1985 and 1997 who were diagnosed with an International Classification of Diseases, 10^th^ Revision (World Health Organization, 1992) (ICD-10) non-affective (F20–29) or affective psychotic disorder (F30.2, F31.2, F31.5, F32.3, F33.3) in the National Patient Register (NPR) for the first time after their 16^th^ birthday, followed until 31 December 2016. The NPR has recorded all inpatient admissions since 1973, and began capturing outpatient care and admission type (voluntary/involuntary) in 2001, and is known to be reliable and valid for research purposes (Dalman, Broms, Cullberg, & Allebeck, 2002). It is known to provide complete coverage of in-patient care in Sweden for the entire period of our study, with complete out-patient care since 2006 (Jörgensen, Ahlbom, Allebeck, & Dalman, 2010). We excluded individuals with a recorded diagnosis of the psychotic disorder before their 16^th^ birthday (*N* = 443). All individuals were official residents of Sweden at diagnosis. Individuals without permanent residency in Sweden (i.e. temporary visitors, undocumented migrants and asylum seekers) were not included in this study.

### Outcome

Our primary outcome was compulsory admission status at the time of the first diagnosis of psychotic disorder. This is recorded in the NPR for each contact with health services (in- or out-patient) under the Compulsory Psychiatric Care Act (Dressing & Salize, 2004), as a binary variable for compulsory or voluntary admission. For individuals with multiple health service admissions on the same day, evidence of compulsory admission took precedence.

### Individual-level variables

Our primary exposure was migrant status, defined according to combined information held in the national immigration and emigration register (STATIV) and the multigenerational register. We categorised individuals into three migrant groups: *Swedish-born*, defined as those born in Sweden to two Swedish-born parents; *migrants*, defined as people born outside of Sweden who later immigrated, and; *children of migrants*, defined as those born in Sweden to at least one foreign-born parent, consistent with an earlier study (Dykxhoorn et al., 2018).

As a second exposure variable, we defined a ‘region-of-origin’ variable to further categorise individuals into seven broad regions (online Supplementary Table S1): Sweden, other Nordic countries, other European countries, Northern Africa and the Middle East, sub-Saharan Africa, Asia and Oceania, and North and South America (Dykxhoorn et al., 2018). Children of migrants whose parents both came from the same region were assigned to that region-of-origin. Children of migrants whose parents came from different regions-of-origin were recorded as either having mixed *Swedish-Nordic* heritage (23% of all children of migrants in the present sample), *Swedish-migrant* heritage (32.9% of all children of migrants) or other *mixed migrant* heritage (6.4% of all children of migrants).

We considered several potential confounders in our models. Age-at-diagnosis (16–19, 20–23, 24–27, 28–31 years) and sex were recorded from the RTP and NPR at time of first psychotic disorder diagnosis. Disposable family income as a potential confounder was obtained from the Longitudinal Integration Database for Health Insurance and Labour Market Studies (LISA), which records total disposable family income from all sources each year (salary, wages, welfare, pensions), weighted for family size, with younger children receiving less weight. In each year, we divided family income for all Swedish residents into quintiles, implicitly accounting for inflation, and assigned individuals to their family disposable income quintile in the year of their 15^th^ birthday. Individuals who immigrated to Sweden after age 15 were assigned their first recorded family income quintile.

### Neighbourhood-level variables

We obtained the Small Area Market Statistics (SAMS) neighbourhood that individuals were living in their year of diagnosis from the RTP. SAMS areas contain between 1000 and 2000 individuals and are devised by Statistics Sweden based on election districts in more rural areas, and by dividing cities into neighbourhoods of socioeconomically homogenous housing (Sundquist, Malmström, & Johansson, 2004). We merged any SAMS areas with less than 50 inhabitants with an adjacent SAMS area to create 7416 neighbourhoods. Our cohort came from 4144 (55.5%) of these SAMS areas.

For each SAMS, in each year, we estimated population density as people per square kilometre. Likewise, we estimated SAMS neighbourhood deprivation, based on four indicators obtained from the LISA, including the proportion of people who were: unemployed; receiving social welfare; with income below the median national income, or; having a criminal conviction in each SAMS per year. These indicators were z-standardised and summed to create a continuous measure of deprivation, where higher scores represented more deprived areas. We also estimated an equivalent of own-group neighbourhood-level ethnic density in the Swedish context, based on region-of-origin, because ethnicity is not routinely recorded in Swedish register data. Henceforth, ‘own-region migrant density’ was estimated separately for people for each region-of-origin described above (except the Swedish-born), as the proportion of people from each region-of-origin as a function of the total population of each SAMS in a given year. Migrants and children of migrants were assigned their own-region migrant density, with children of migrants from mixed backgrounds being assigned either as of Swedish-migrant or mixed-migrant origin, except those of Swedish-Nordic heritage who were assigned Nordic migrant density values in these analyses. All neighbourhood-level covariates (population density, deprivation and own-region migrant density) were *z*-standardised to have a mean of zero and standard deviation of one for modelling purposes. We also created quintiles of each variable for descriptive characterisation of the sample.

### Statistical analysis

We compared the distribution of compulsory admission, demographic and neighbourhood-level characteristics by migrant status using percentages and Pearson's chi-square tests. Next, we fitted unadjusted and multivariable multilevel logistic regression models to examine the effect of migrant status, region-of-origin and own-region migrant density on risk of compulsory admission. Multilevel models were fitted with random intercepts at the neighbourhood (SAMS) level to take into account the nested structure of the data (individuals within neighbourhoods) and to quantify variation in compulsory admission attributable to the neighbourhood-level, in null (i.e. outcome-only) and fully adjusted models, expressed as intraclass correlation coefficients (ICC).

Modelling proceeded as follows. First, we reported unadjusted odds ratios (OR) to characterise their univariable association with compulsory admission at first diagnosis of psychotic disorder. Second, we fitted a multivariable model with age and sex as *a priori* confounders to examine the effect of migrant status on compulsory admission. Other confounders (family income, population density, deprivation) were added to the model in order of the strength of their univariable associations (estimated via Akaike's Information Criterion), which we retained if they improved model fit, assessed via a likelihood ratio test (LRT). Next, we added region-of-origin to our model to examine whether this provided a better fit of the data than migrant status, assessed via LRT and reported effect sizes by region-of-origin if appropriate. Third, to examine whether own-region migrant density was associated with the risk of compulsory admission we repeated the analyses above on a sample restricted to migrants and children of migrants (i.e. excluding the Swedish-born sample). Finally, we examined whether region-of-origin, own-region migrant density, population density and deprivation had differential effects for migrants *v.* children of migrants by inspecting stratified models and reporting LRT *p* values for effect modification between migrant status and these variables on the risk of compulsory admission. Analyses were based on complete case analysis, with minimal missing data (*N* = 360; 3.0%). OR were presented with 95% confidence intervals (95%CI) and statistical significance set at *p* < 0.05. OR for compulsory admission associated with all neighbourhood variables were expressed as the OR for a one standard deviation change (increase) in the exposure. All modelling was conducted in Stata 14.

## Results

### Sample characteristics

Our cohort included 12 000 individuals born between 1985 and 1997 and diagnosed with a psychotic disorder in Sweden after their 16^th^ birthday. One individual was missing data on the migrant status and another on region-of-origin (0.02%). There were also 165 (1.4%) individuals missing data on neighbourhood population density and deprivation, 92 (0.8%) on own-region migrant density and 201 (1.7%) on family income (Table 1). The complete case sample for analysis included 11 640 (97%) of the cohort. Individuals with missing data differed from the analytical sample on all variables except population density quintile (χ^2^ *p* = 0.12; online Supplementary Table S2), and, in particular, were more likely to have been involuntarily admitted (4.0% *v.* 3.0% missing in individuals with voluntary admission; χ^2^ *p* = 0.02) and be migrants (10.7% *v.* 1.6% and 1.2% missing in children of migrants and Swedish-born individuals, respectively; χ^2^ *p* < 0.001).

**Table 1.** Cohort characteristics by migrant status (*N* = 11 999)^a^

	Migrants		Children of migrants		Swedish-born		χ^2^-test (df);
Variables	*n*	%	*n*	%	*n*	%	*p* value
Compulsory admission							31.3 (2); <0.001
Voluntary	1853	86.5	2343	88.0	6505	90.4	
Involuntary	289	13.5	320	12.0	689	9.6	
Sex							29.7 (2); <0.001
Male	1358	63.4	1647	61.8	4148	57.7	
Female	784	36.6	1016	38.2	3046	42.3	
Age at diagnosis							64.5 (6); <0.001
16–19	482	22.5	780	29.3	2039	28.3	
20–23	830	38.8	1084	40.7	2810	39.1	
24–27	599	28.0	612	23.0	1809	25.1	
28–31	231	10.8	187	7.0	536	7.5	
Region								
Sweden	–	–	–	–	7194	100.0	–
Nordic	64	3.0	182	6.8	–	–	
Europe	578	27.0	214	8.0	–	–	
Asia + Oceania	328	15.3	61	2.3	–	–	
Middle East + North Africa	555	25.9	335	12.6	–	–	
Sub-Saharan Africa	445	20.8	129	4.8	–	–	
North + South America	171	8.0	71	2.7	–	–	
Children of migrants:								
Swedish-Nordic	–	–	629	23.6	–	–	
Swedish-migrant	–	–	872	32.8	–	–	
Mixed migrant	–	–	170	6.4	–	–	
Missing	–	–	1	0.04	–	–	
Family income							2376.8 (8); <0.001
Quintile 1 (lowest)	720	33.6	219	8.2	386	5.4	
Quintile 2	302	14.1	594	22.3	1234	17.2	
Quintile 3	407	19.0	778	29.2	1570	21.8	
Quintile 4	319	14.9	624	23.4	2067	28.7	
Quintile 5 (highest)	218	10.2	438	16.5	1922	26.7	
Missing	176	8.2	10	0.4	15	0.2	
Population density							574.0 (8); <0.001
Quintile 1 (lowest)	143	6.7	237	9.0	1228	17.2	
Quintile 2	253	11.8	346	13.2	1387	19.5	
Quintile 3	344	16.1	442	16.8	1450	20.3	
Quintile 4	607	28.3	664	25.2	1544	21.7	
Quintile 5 (highest)	735	34.3	942	35.8	1512	21.2	
Missing	60	2.8	32	1.2	73	1.0	
Deprivation index							710.2 (8); <0.001
Quintile 1 (lowest)	172	8.0	281	10.6	983	13.7	
Quintile 2	167	7.8	357	13.4	1230	17.1	
Quintile 3	238	11.1	410	15.4	1410	19.6	
Quintile 4	389	18.2	512	19.2	1696	23.6	
Quintile 5 (highest)	1116	52.1	1071	40.2	1802	25.1	
Missing	60	2.8	32	1.2	73	1.0	
Own-region migrant density							226.3 (8); <0.001
Quintile 1 (lowest)	551	25.7	392	14.7	–	–	
Quintile 2	342	16.0	601	22.6	–	–	
Quintile 3	283	13.2	659	24.8	–	–	
Quintile 4	382	17.8	562	21.1	–	–	
Quintile 5 (highest)	524	24.5	417	15.7	–	–	
Missing	60	2.8	32	1.2	–	–	

a Excluding one person with missing migrant status.

In total, 1298 (10.8%) individuals were admitted compulsorily at their first admission for psychotic disorder, a figure that was higher in migrants (13.5%) than their children (12.0%) or the Swedish-born population (9.6%; χ^2^ *p* < 0.001). Median age at first diagnosis was 22.2 years old [interquartile range (IQR): 19.8–25.1; range: 16.0–31.8], though this differed between children of migrants (21.9 years; IQR: 19.6–24.6), Swedish-born (22.1; IQR: 19.7–25.0) and migrant (22.9; IQR: 20.4–25.6) individuals (Kruskal–Wallis χ^2^ test *p* < 0.001; online Supplementary Fig. S1). Most of the sample were male (59.6%) and Swedish-born (60.0%). Individuals were over-represented in more densely populated and deprived neighbourhood quintiles, although this was more pronounced amongst migrants and children of migrants (Table 1; both χ^2^ *p* < 0.001). Migrants were also more likely to be in lower-income quintiles than children of migrants and the Swedish-born population (χ^2^ *p* < 0.001). Migrants were most likely to come from non-Nordic European countries (27.0%), the Middle East and North Africa (25.9%) or sub-Saharan Africa (20.8%), while children of migrants were predominantly of dual Swedish-migrant (32.8%), Swedish-Nordic origin (23.6%) or Middle Eastern and North African origin (12.6%).

### Migrant status and risk of compulsory admission

A null multilevel logistic model indicated that about 5.5% (95% CI 2.9–10.3) of variance in compulsory admission could be attributed to neighbourhood level effects (*p* < 0.001). These remained statistically significant in the final adjusted model (Table 2; ICC: 3.5%; 95% CI 1.2–9.5; *p* = 0.02).

**Table 2.** Unadjusted and adjusted odds of compulsory admission (*n* = 11 640)

	Compulsory admission		Unadjusted			Adjusted^a^		
	*n*	%	OR	95% CI		OR	95% CI	
Migrant status								
Swedish-born	675	9.5	1.00	–	–	–	–	–
Migrants	259	13.6	**1.48**	**1.26**	**1.73**	–	–	–
Children of migrants	312	11.9	**1.27**	**1.10**	**1.47**	–	–	–
Sex								
Female	429	9.1	1.00	–	–	1.00	–	–
Male	817	11.8	**1.34**	**1.19**	**1.52**	**1.28**	**1.13**	**1.45**
Age at diagnosis								
16–19	266	8.2	1.00	–	–	1.00	–	–
20–23	524	11.5	**1.47**	**1.25**	**1.72**	**1.41**	**1.20**	**1.65**
24–27	311	10.6	**1.35**	**1.13**	**1.60**	**1.28**	**1.07**	**1.52**
28–31	145	15.9	**2.14**	**1.71**	**2.67**	**2.02**	**1.61**	**2.52**
Region								
Sweden	675	9.5	1.00	–	–	1.00	–	–
Nordic	16	6.8	0.69	0.41	1.16	0.68	0.40	1.14
Europe	93	12.4	**1.35**	**1.07**	**1.71**	**1.27**	**1.01**	**1.61**
Asia + Oceania	40	11.7	1.20	0.85	1.69	1.17	0.83	1.65
Middle East + North Africa	118	14.4	**1.58**	**1.27**	**1.96**	**1.46**	**1.17**	**1.81**
Sub-Saharan Africa	91	18.0	**2.06**	**1.61**	**2.65**	**1.94**	**1.51**	**2.49**
North + South America	29	12.5	1.36	0.91	2.04	1.29	0.86	1.93
*Children of migrants:*								
Swedish-Nordic	75	12.3	**1.35**	**1.04**	**1.75**	**1.33**	**1.03**	**1.72**
Swedish-migrant	95	11.0	1.17	0.93	1.48	1.13	0.89	1.42
Mixed migrant	14	8.3	0.83	0.48	1.46	0.79	0.45	1.39
Family income								
Quintile 1 (lowest)	151	11.8	1.00	–	–	–	–	–
Quintile 2	240	11.4	0.97	0.78	1.22	–	–	–
Quintile 3	281	10.4	0.87	0.70	1.08	–	–	–
Quintile 4	309	10.4	0.88	0.71	1.08	–	–	–
Quintile 5 (highest)	265	10.3	0.88	0.70	1.09	–	–	–
Population density (*z*-standardised)^b^	–	–	**1.14**	**1.08**	**1.21**	**1.12**	**1.06**	**1.18**
Neighbourhood deprivation (*z*-standardised)^b^	–	–	**1.13**	**1.07**	**1.20**	–	–	–
Intraclass correlation coefficient (%)^c^			**0.05**	**0.03**	**0.10**	**0.04**	**0.01**	**0.10**

Bold denotes *p*<0.05

a Adjusted for sex, age group at diagnosis, region of origin and population density. No other variables improved the final model fit.

b Fitted data are better as continuous variables.

c As reported in null (*p* < 0.01) and fully adjusted models (*p* = 0.02).

In an unadjusted model, being a migrant (OR 1.48, 95% CI 1.26–1.73) or child of a migrant (OR 1.27, 95% CI 1.10–1.47) was associated with increased risk of compulsory admission compared with the Swedish-born population (Table 2). Being male, older and from a more deprived and densely populated neighbourhood were also associated with increased risk of compulsory admission in univariable models. In our final model, region-of-origin provided a better fit to the data than migrant status. Thus, after adjustment for age, sex and population density, we observed increased risk of compulsory admission in individuals from the Middle East and North Africa (adjusted OR 1.46, 95% CI 1.17–1.81), sub-Saharan Africa (adjusted OR 1.94, 95% CI 1.51–2.49) and non-Nordic Europe (OR 1.27; 95% CI 1.01–1.61), as well as in Swedish-Nordic children of migrants (adjusted OR 1.33; 95% CI 1.03–1.72) compared with the Swedish-born group. These patterns were unaltered after additional adjustment for family disposable income (online Supplementary Table S3).

Male sex and older age at first diagnosis were associated with increased risk of compulsory admission in our adjusted model (Table 2), as well as individuals from more densely populated neighbourhoods (per standard deviation increase in population density: adjusted OR 1.12, 95% CI 1.06–1.19).

### Own-region migrant density and compulsory admission

We restricted our analysis of the effect of own-region migrant density on risk of compulsory admission to migrants and children of migrants (*N* = 4533). In univariable models, greater population density, deprivation and migrant density were all associated with increased risk of compulsory admission in this sample (Table 3). After multivariable modelling, only greater own-region migrant density remained associated with compulsory admission (per standard deviation increase in own-region migrant density: adjusted OR 1.12; 95% CI 1.02–1.24), adjusted for age group, sex, region-of-origin and population density.

**Table 3.** Unadjusted and adjusted odds of compulsory admission for migrants and children of migrants (*n* = 4533)

	Compulsory admission		Unadjusted			Adjusted		
	*n*	%	OR	95% CI		OR^a^	95% CI	
Migrant status								
Migrants	259	13.6	1.00	–	–	–	–	–
Children of migrants	312	11.9	0.86	0.72	1.03	–	–	–
Sex								
Female	182	10.6	1.00	–	–	1.00	–	–
Male	389	13.8	**1.35**	**1.11**	**1.64**	**1.31**	**1.08**	**1.59**
Age at diagnosis								
16–19	124	10.2	1.00	–	–	1.00	–	–
20–23	249	14.0	**1.46**	**1.15**	**1.84**	**1.39**	**1.10**	**1.76**
24–27	130	11.4	1.15	0.88	1.50	1.13	0.86	1.48
28–31	68	17.4	**1.90**	**1.37**	**2.65**	**1.81**	**1.30**	**2.53**
Region								
Nordic	16	6.8	1.00	–	–	1.00	–	–
Europe	93	12.4	**1.99**	**1.13**	**3.48**	**1.80**	**1.02**	**3.16**
Asia + Oceania	40	11.7	1.74	0.94	3.22	1.73	0.92	3.23
Middle East + North Africa	118	14.4	**1.31**	**1.33**	**4.02**	**1.98**	**1.13**	**3.49**
Sub-Saharan Africa	91	18.0	**3.04**	**1.73**	**5.37**	**2.90**	**1.64**	**5.14**
North + South America	29	12.5	**2.00**	**1.04**	**3.84**	**2.06**	**1.07**	**3.97**
*Children of migrants:*								
Swedish-Nordic	75	12.3	**1.97**	**1.11**	**3.48**	**2.03**	**1.14**	**3.61**
Swedish-migrant	95	11.0	1.73	0.99	3.02	1.73	0.99	3.04
Mixed migrant	14	8.3	1.21	0.57	2.59	1.25	0.59	2.69
Family income								
Quintile 1 (lowest)	115	12.7	1.00	–	–	–	–	–
Quintile 2	121	13.8	1.11	0.84	1.46	–	–	–
Quintile 3	134	11.5	0.90	0.68	1.18	–	–	–
Quintile 4	128	13.7	1.10	0.83	1.45	–	–	–
Quintile 5 (highest)	73	11.2	0.87	0.64	1.20	–	–	–
Population density (*z*-standardised)	–	–	**1.12**	**1.03**	**1.22**	1.08	0.99	1.18
Neighbourhood deprivation (*z*-standardised)	–	–	**1.12**	**1.04**	**1.20**	–	–	–
Neighbourhood own-region migrant density (*z*-standardised)			**1.16**	**1.07**	**1.25**	**1.12**	**1.02**	**1.24**
Intraclass correlation coefficient (%)^b^			**0.06**	**0.02**	**0.15**	**0.04**	**0.01**	**0.19**

a Adjusted for sex, age group at diagnosis, region of origin and population density. No other variables improved the final model fit.

b As reported in null (*p* = 0.02) and fully adjusted models (*p* = 0.10).

In observing data during primary analyses, a potential differential effect of region of origin on compulsory admission between migrants and children of migrants emerged, and we conducted post-hoc analyses to establish significance. However, there was no evidence that the effect of region-of-origin (LRT *p* = 0.97), population density (LRT *p* = 0.55), or own-region migrant density (LRT *p* = 0.47) on compulsory admission differed between migrants and children of migrants, although the effects of region-of-origin and own-region migrant density appeared more robust to statistical chance in children of migrants (online Supplementary Table S4).

## Discussion

### Main findings

In a large, population-based nationwide sample of people experiencing their first admission for psychosis in Sweden, we found evidence that migrants and children of migrants were more likely to be compulsorily admitted at first admission for psychotic disorder than the Swedish-born population, consistent with most evidence (Cole et al., 1995; Davies et al., 1996; Fassaert et al., 2016; Mann et al., 2014; Morgan et al., 2005; Mulder et al., 2006; Rotenberg et al., 2017; Singh et al., 2015). Our models demonstrated that this risk was more strongly patterned by region-of-origin than migrant status, with individuals from sub-Saharan African, Middle Eastern and North African, and non-Nordic European backgrounds at increased risk of compulsory admission for psychotic disorder. Interestingly, compared with Swedish-born individuals, born to two Swedish-born parents, children of migrants from mixed Swedish-Nordic backgrounds were also more likely to be compulsorily admitted at first admission for a psychotic disorder. These findings were independent of age, gender, family income at age 15 or neighbourhood-level deprivation and population density, although the latter was independently associated with greater risk of compulsory admission for a psychotic disorder. We also found evidence that higher neighbourhood own-region migrant density was associated with an increased risk of compulsory admission in migrants and children of migrants. Taken together, these findings suggest that cultural and structural barriers over and above the effects of socioeconomic factors may affect the risk of compulsory admission for psychotic disorder in Sweden.

### Strengths and limitations

Our study used longitudinal data from Sweden's national registers, allowing us to access information on all adults diagnosed with a psychotic disorder for the first time in Sweden, with minimal missing data. While those individuals missing data differed significantly from our analytical sample on a number of variables, the small amount of missing data in our sample (3%) decreased the likelihood that these differential patterns of missingness had substantial import on our results. The Swedish NPR began recording involuntary admission status in 2001, giving us up to 15 years of follow-up data on this sample with almost complete coverage of psychiatric care over this period (Jörgensen et al., 2010). We do not believe the less than 100% coverage of outpatient care between 2001 and 2005 would have had differential effects by migrant status. Despite the large sample size, we may have lacked the power to detect true variation in compulsory admission for some groups (particularly those from Nordic regions and the Americas). This routinely collected dataset was prospectively recorded, minimising recall bias. Our large sample size allowed us to estimate relatively precise effect sizes for compulsory admission at first admission for psychosis on a range of individual- and neighbourhood-level factors. We reasoned that neighbourhood-level factors measured in the same year as the first diagnosis would be more relevant to someone's pathway to care than earlier neighbourhood level exposures. Sweden does not record ethnicity in its registers, so we were reliant on region-of-origin to estimate both individual-level risk and associations with migrant density. We acknowledge that in relying on broad regions-of-origin, we may have grouped together heterogeneous groups in terms of their ethnic or cultural background, or their social or economic experience of migration, occluding our ability to isolate the reasons for certain groups' elevated risk of compulsory admission. We did not adjust our analyses for diagnosis type (i.e. non-affective *v.* affective), which was recorded in the NPR based on clinical discharge diagnoses made downstream of our outcome (admission status); this may therefore not have been independent of outcome status, potentially introducing bias. We were also limited in being able to understand potential legal barriers to care and their implications for the risk of compulsory admission, as the Swedish registers do not include data on those living in the country without permanent residency. An additional limitation of using register-based was our reliance on available variables; we were unable to include data on potentially relevant factors such as symptom severity at presentation, grounds for a compulsory admission decision, or access to healthcare services prior to the first admission. Nonetheless, such challenges are common to all register-based research, and the variation in compulsory admission we observed could not be attributed to demographic differences, family income or major neighbourhood-level socioenvironmental factors, which should inform current theories for why many migrant and ethnic minority groups face an elevated risk of compulsory admission for psychiatric care.

### Implications of the research

The prevalence of compulsory admission at first diagnosis of psychotic disorder in this nationwide Swedish sample (10.5%) was lower than observed elsewhere. For example, compulsory admission in first-episode psychosis samples has been reported to be between 19% and 29% in the Netherlands (Fassaert et al., 2016; Mulder et al., 2006), 31%–38% in London (Cole et al., 1995; Morgan et al., 2005) and 32% in Toronto (Rotenberg et al., 2017). Several reasons to explain this discrepancy are likely to exist. Most parsimoniously, we suggest it is explained by possible variation in national legal frameworks, policies and practices which result in differential criteria for, and usage of, involuntary detention; even within Europe differences in implementation of early intervention for psychosis care or other innovative treatment programs may partially account for variable rates of involuntary detention (de Stefano & Ducci, 2008). In neighbouring Denmark, for example, which like Sweden is one of only a handful of countries where the final decision on compulsory detention is made by a psychiatrist, the nationwide prevalence of involuntary detention for any psychiatric disorder is around 7% in the native Danish population (Norredam, Garcia-Lopez, Keiding, & Krasnik, 2010).

More widely, our results demonstrated the importance of considering region-of-origin, over and above migrant status, to understand patterns of compulsory admission in Sweden. This finding suggests that the shared experience of migration may be less salient to an elevated risk of involuntary detention for psychotic disorder than cultural or structural forces which are specific to certain migrant populations. Here, increased risk of compulsory admission was largely concentrated in migrants from specific regions, namely the Middle East and Africa. This pattern has also been shown in studies from the UK, Canada and Netherlands pointing towards the prominence of group-specific structures over the country of residence specific contexts (Morgan et al., 2005; Mulder et al., 2006; Rotenberg et al., 2017; Thomas et al., 1993; Weich et al., 2017). It has been suggested that experiences of structural or institutionalised racism may contribute to disproportionate rates of involuntary admission in some migrant and minority groups (Davies et al., 1996; McKenzie & Bhui, 2007), and while our study could not directly investigate this, we noted that exact risk of compulsory admission amongst migrants by region-of-origin was approximately correlated with the likely visible minority status of those migrants. Migrant groups may also face additional barriers to accessing services including language and healthcare literacy. This hypothesis is supported by the higher rates of compulsory admission found in neighbourhoods with higher own-region migrant density, suggesting that there may be concentrated cultural factors which affect access to timely mental health care in these communities. This information will be useful for policymakers and service providers to reduce compulsory pathways to care in potentially difficult to reach or underserved communities in Sweden.

Nonetheless, the raised risk of compulsory admission amongst non-Nordic European migrants and children of migrants from mixed Swedish-Nordic parentage challenges the hypothesis that variation in rates of compulsory admission by region-of-origin is entirely attributable to cultural factors. For example, these groups may have fewer differences in culture-bound ideas of mental health compared with the Swedish-born population, a factor which has been hypothesised to delay seeking care, thus increasing the risk of compulsory admission. Interestingly, although structural differences in income patterns existed between migrant groups and the Swedish-born reference population (online Supplementary Table S5), this did not account for the excess risk of compulsory admission for people of mixed Swedish-Nordic heritage or other European backgrounds (online Supplementary Table S3). This suggests further research on post-migratory pathways to care for psychosis in different migrant groups appears warranted to contextualise these patterns.

In England, Weich et al. (2017) identified a similar proportion of variance in compulsory admission (for all psychiatric disorders) could be attributed to small area neighbourhood factors, as observed in our paper. However, in their study deprivation, not population density, was a stronger predictor of compulsory admission, in contrast to our findings for psychosis. We found that living in more densely populated areas was associated with increased risk of compulsory admission for psychotic disorder, which may expose people to more chaotic or stressful environments close to the onset or to socially isolated neighbourhoods where they lack immediate support from family or friends which might otherwise mitigate the need for compulsory care. Corollary evidence suggests that people living in urban areas may experience longer delays in seeking treatment for care (Boonstra et al., 2012) and that the duration of untreated psychosis is longer for people living in more socially fragmented (although not more densely populated (Kirkbride et al., 2010)) neighbourhoods (O'Donoghue et al., 2016).

For migrants and children of migrants, we also found that higher own-region migrant density was associated with a higher risk of compulsory admission, independent of population density. This finding suggests that culture-bound understanding of mental health (Sharpley et al., 2001) or patterns of structural exclusion (Keating et al., 2002; McKenzie & Bhui, 2007; Singh & Burns, 2006) may interact with the use of the Swedish healthcare system to increase risk of compulsory admission, although a dearth of literature on this important issue remains evident (Sundquist, 2001). This may take various forms, including delayed first contact with services for psychosis which may increase the likelihood that their presentation will be judged to require involuntary care. Such delayed contact might arise due to lower healthcare literacy, language difficulties, distrust of institutionalised care, under-provision of culturally appropriate care, or use of traditional or alternative methods to provide care for people with mental health difficulties (Keating et al., 2002; McKenzie & Bhui, 2007; Sharpley et al., 2001; Singh & Burns, 2006); future investigation of the specific structural and cultural factors which may drive ongoing injustices in compulsory care faced by migrant and minority groups is now vital.

We have shown that migrants and children of migrants living in Sweden, and coming from several regions-of-origin, are at increased risk of compulsory admission for their first diagnosis of psychotic disorder, drawing unenviable parallels with findings from the UK, the Netherlands and Canada. Our results provide further contextualization of these issues and highlight the likely interactions between cultural, socio-demographic and structural barriers which may shape variation in risk of compulsory admission for care for psychotic disorders. This phenomenon requires an international research response to elucidate the qualitative mechanisms that maintain this inequality so we can provide more equitable access, pathways and treatment for first-epsiode psychosis, regardless of race, ethnicity or migrant status.

## Supplementary material

For supplementary material accompanying this paper visit https://doi.org/10.1017/S0033291720002068. click here to view supplementary material
