# Supplementary material for: Migrant status and risk of compulsory admission at first diagnosis of psychotic disorder: a population-based cohort study in Sweden
Source: Psychol Med. 2020 Jun 24;52(2):362–71. doi: 10.1017/S0033291720002068 (PMC8842197; doi:10.1017/S0033291720002068)
Supplement: Supplementary file 1 [file S0033291720002068sup001.docx]

***
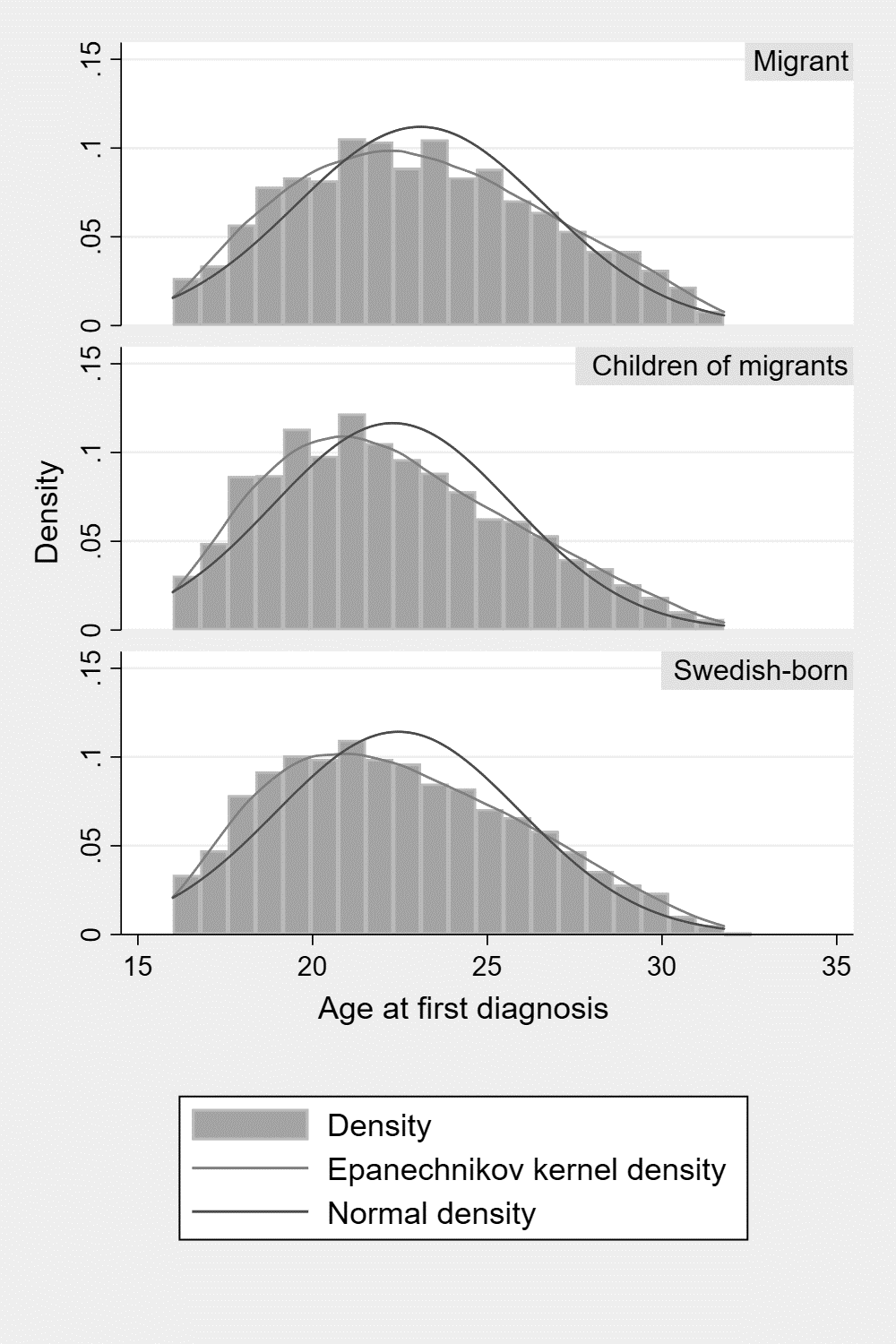
*Supplemental Figure 1: Histogram of age at first diagnosis, by migrant status**

**Legend**: The distribution of age-at-first diagnosis for psychotic disorder was positively skewed for all groups (see blue and green density functions), but differed by migrant status (Kruskal-Wallis Χ^2^ test on 2 degrees of freedom: 66.7; p<0.001), with migrants having a later median age-at-first-diagnosis (22.9; IQR: 20.4-25.6) than children of migrants (21.9 years; IQR: 19.6-24.6) or Swedish-born participants (22.1; IQR: 19.7-25.0).

**Supplement Table 1: List of countries by region of origin**

| **Region of origin** | **SMA classification** | **Countries^1^** |
| --- | --- | --- |
|  |  |  |
| Sweden^2^ | Sweden | Sweden |
| Nordic | Nordic | Denmark, Norway, Finland, Iceland |
| Europe | UK & Ireland | UK, Ireland |
|  | Western Europe | Austria, Belgium, France, Germany, Liechtenstein, Luxembourg, Netherlands, Switzerland |
|  | Southern Europe | Andorra, Gibraltar, Greece, Italy, Malta, Monaco, Portugal, San Marino, Spain, Vatican City |
|  | Eastern Europe | Albania, Belarus, Bulgaria, Czech Republic, Hungary, Moldova, Poland, Romania, Slovakia, Ukraine |
|  | Former Yugoslavia | Bosnia Herzegovina, Croatia, Kosovo, Macedonia, Montenegro, Serbia, Slovenia |
|  | Russia & the Baltic States | Estonia, Latvia, Lithuania, Russia |
| Asia & Oceania | Oceania | Australia, New Zealand, Fiji, Kiribati, Marshall Islands, Micronesia Federated States, Nauru, Palau, Papua New Guinea, Samoa, Solomon Islands, Tuvalu, Tonga, Vanuatu |
|  | Central Asia | Afghanistan, Armenia, Azerbaijan, Bangladesh, Bhutan, Georgia, India, Kazakhstan, Kyrgyzstan, Maldives, Nepal, Pakistan, Sri Lanka, Tajikistan, Turkmenistan |
|  | Northeast Asia | China, Japan, Mongolia, People’s Republic of Korea, South Korea, Taiwan |
|  | Southeast Asia | Brunei, Cambodia, East Timor, Hong Kong, Indonesia, Laos, Malaysia, Myanmar, Philippines, Singapore, Thailand, Vietnam |
| Middle East & North African | Iran | Iran |
|  | Iraq | Iraq |
|  | Middle East, other | Bahrain, Cyprus, Israel, Jordan, Kuwait, Lebanon, Oman, Palestine, Qatar, Saudi Arabia, Syria, United Arab Emirates, Yemen, Turkey |
|  | North Africa | Algeria, Egypt, Libya, Morocco, Tunisia |
| Sub-Saharan Africa | West Africa | Benin, Burkina Faso, Cape Verde, Gambia, Ghana, Guinea, Guinea Bissau, Ivory Coast, Liberia, Mali, Mauritania, Niger, Nigeria, Senegal, Sierra Leone, Togo |
|  | East Africa | Djibouti, Eritrea, Ethiopia ,Somalia |
|  | Africa, other | Angola, Botswana, Burundi, Cameroon, Central African Republic, Chad, Comoros, Congo, Democratic Republic of Congo, Equatorial Guinea, Gabon, Kenya, Lesotho, Madagascar, Malawi, Mauritius, Mozambique, Namibia, Rwanda, Sao Tome and Principe, Seychelles, South Africa, Swaziland, Tanzania, Uganda, Zambia, Zanzibar |
| North & South America | USA & Canada | USA, Canada |
|  | North America, other | Antigua and Barbuda, Bahamas, Barbados, Belize, Costa Rica, Cuba, Dominica, Dominican Republic, El Salvador, Grenada, Guatemala, Haiti, Honduras, Jamaica, Mexico, Nicaragua, Panama, Saint Kitts and Nevis, Saint Lucia, Saint Vincent and the Grenadines, Trinidad and Tobago |
|  | South America, South | Argentina, Brasil, Chile, Uruguay |
|  | South America, other | Bolivia, Colombia, Ecuador, Guyana, Paraguay, Peru, Surinam, Venezuela |

^1^Countries of origin, as defined by the Swedish Migration Agency [SMA], in each SMA category. Country-level data is not made available by the SMA/Statistics Sweden for research purposes.

**Supplement Table 2: Comparison of sample by missing data status**

| Variables | Non-missing | | Missing | | Χ^2^-test (df); |  |
| --- | --- | --- | --- | --- | --- | --- |
|  | **n** | **%** | **n** | **%** | **p-value** | |
| Compulsory admission |  |  |  |  | 5.1 (1); 0.02 | |
| Voluntary | 10,394 | 97.1 | 308 | 3.0 |  | |
| Involuntary | 1,246 | 96.0 | 52 | 4.0 |  | |
| Migrant status |  |  |  |  | 539.9 (2); <0.001 | |
| Swedish-born | 7,107 | 98.8 | 87 | 1.2 |  | |
| Migrants | 1,912 | 89.3 | 230 | 10.7 |  | |
| Children of migrants | 2,621 | 98.4 | 42 | 1.6 |  | |
| Sex |  |  |  |  | 8.3 (1); 0.004 | |
| Male | 6,913 | 96.6 | 241 | 3.4 |  | |
| Female | 4,727 | 97.5 | 119 | 2.5 |  | |
| Age at diagnosis |  |  |  |  | 31.0 (3); <0.001 | |
| 16-19 | 3,246 | 98.3 | 56 | 1.7 |  | |
| 20-23 | 4,559 | 96.5 | 165 | 3.5 |  | |
| 24-27 | 2,924 | 96.8 | 96 | 3.2 |  | |
| 28-31 | 911 | 95.5 | 43 | 4.5 |  | |
| Region |  |  |  |  | 398.7 (9); <0.001 | |
| Sweden | 7,107 | 98.8 | 87 | 1.2 | - | |
| Nordic | 235 | 95.5 | 11 | 4.5 |  | |
| Europe | 747 | 94.3 | 45 | 5.7 |  | |
| Asia + Oceania | 355 | 91.3 | 34 | 8.7 |  | |
| Middle East + North Africa | 817 | 91.8 | 73 | 8.2 |  | |
| Sub-Saharan Africa | 506 | 88.2 | 68 | 11.9 |  | |
| North + South America | 232 | 95.9 | 10 | 4.1 |  | |
| *Children of migrants:* |  |  |  |  |  | |
| Swedish-Nordic | 610 | 97.0 | 19 | 3.0 |  | |
| Swedish-migrant | 862 | 98.9 | 10 | 1.2 |  | |
| Mixed migrant | 169 | 99.4 | 10 | 0.6 |  | |
| Family income |  |  |  |  | 58.5 (4); <0.001 | |
| Quintile 1 (lowest) | 1,281 | 96.7 | 44 | 3.3 |  | |
| Quintile 2 | 2,098 | 98.5 | 32 | 1.5 |  | |
| Quintile 3 | 2,712 | 98.4 | 43 | 1.6 |  | |
| Quintile 4 | 2,983 | 99.1 | 27 | 0.9 |  | |
| Quintile 5 (highest) | 2,566 | 99.5 | 13 | 0.5 |  | |
| Population density |  |  |  |  | 7.3 (4); 0.12 | |
| Quintile 1 (lowest) | 1,594 | 99.1 | 14 | 0.9 |  | |
| Quintile 2 | 1,952 | 98.3 | 34 | 1.7 |  | |
| Quintile 3 | 2,197 | 98.2 | 40 | 1.8 |  | |
| Quintile 4 | 2,762 | 98.1 | 53 | 1.9 |  | |
| Quintile 5 (highest) | 3,135 | 98.3 | 54 | 1.7 |  | |
| Neighbourhood deprivation index |  |  |  |  | 44.2 (4); <0.001 | |
| Quintile 1 (lowest) | 1,424 | 99.1 | 13 | 0.9 |  | |
| Quintile 2 | 1,740 | 99.2 | 14 | 0.8 |  | |
| Quintile 3 | 2,037 | 99.0 | 21 | 1.0 |  | |
| Quintile 4 | 2,557 | 98.5 | 40 | 1.5 |  | |
| Quintile 5 (highest) | 3,882 | 97.3 | 107 | 2.7 |  | |
| Neighbourhood ethnic density^1^ |  |  |  |  | 9.5 (4); 0.05 | |
| Quintile 1 (lowest) | 963 | 95.8 | 40 | 4.2 |  | |
| Quintile 2 | 995 | 96.0 | 38 | 4.0 |  | |
| Quintile 3 | 915 | 97.1 | 27 | 2.9 |  | |
| Quintile 4 | 917 | 97.1 | 27 | 2.9 |  | |
| Quintile 5 (highest) | 893 | 94.9 | 48 | 5.1 |  | |

^1^Comparison restricted to migrants and children of migrants

**Supplement Table 3: Adjusted odds of compulsory admission by region of origin, with and without adjustment for family income (n=11,640)**

|  | **Adjusted**^1^ | | | **Adjusted**^2^ | | |
| --- | --- | --- | --- | --- | --- | --- |
|  | **OR** | **95% CI** | | **OR** | **95% CI** | |
| **Region** |  |  |  |  |  |  |
| Sweden | 1.00 | - | - | 1.00 | - | - |
| Nordic | 0.68 | 0.40 | 1.14 | 0.68 | 0.40 | 1.14 |
| Europe | **1.27** | **1.01** | **1.61** | **1.29** | **1.02** | **1.64** |
| Asia + Oceania | 1.17 | 0.83 | 1.65 | 1.20 | 0.84 | 1.70 |
| Middle East + North Africa | **1.46** | **1.17** | **1.81** | **1.49** | **1.19** | **1.87** |
| Sub-Saharan Africa | **1.94** | **1.51** | **2.49** | **1.99** | **1.54** | **2.57** |
| North + South America | 1.29 | 0.86 | 1.93 | 1.30 | 0.86 | 1.94 |
| *Children of migrants:* |  |  |  |  |  |  |
| Swedish-Nordic | **1.33** | **1.03** | **1.72** | **1.33** | **1.03** | **1.73** |
| Swedish-migrant | 1.13 | 0.89 | 1.42 | 1.13 | 0.90 | 1.43 |
| Mixed migrant | 0.79 | 0.45 | 1.39 | 0.79 | 0.45 | 1.39 |
| **Family income** |  |  |  |  |  |  |
| Quintile 1 (lowest) | - | - | - | 1.00 | - | - |
| Quintile 2 | - | - | - | 1.15 | 0.91 | 1.44 |
| Quintile 3 | - | - | - | 1.00 | 0.80 | 1.25 |
| Quintile 4 | - | - | - | 1.06 | 0.85 | 1.32 |
| Quintile 5 (highest) | - | - | - | 1.09 | 0.87 | 1.37 |

^1^Adjusted for sex, age group at diagnosis and population density. As per Table 2.

^2^Additional adjustment for family income

**Supplement Table 4: Stratified analysis of compulsory admission and neighbourhood own-group migrant density by migrant status, mutually adjusted for other variables**

| Variables | Adjusted OR (95% CI) for migrants^1^ | Adjusted OR (95% CI) for children of migrants^1^ | LRT (df) p-value for interaction with migrant status^2^ |
| --- | --- | --- | --- |
| Participant N (%): | 1,800 (40.9%) | 2,605 (59.1%) | - |
| Region of origin |  |  | 1.0 (5); p=0.97 |
| Nordic | 1 | 1 |  |
| Europe | 1.25 (0.47 – 3.31) | **2.19 (1.03 – 4.64)** |  |
| Asia + Oceania | 1.24 (0.45 – 3.42) | 1.87 (0.64 – 5.43) |  |
| Middle East + North Africa | 1.45 (0.54 – 3.89) | **2.22 (1.08 – 4.58)** |  |
| Sub-Saharan Africa | 1.99 (0.75 – 5.31) | **3.59 (1.63 – 7.88)** |  |
| North + South America | 1.47 (0.52 – 4.22) | 2.31 (0.86 – 6.22) |  |
| *Children of migrants:* |  |  |  |
| Swedish-Nordic | - | **2.41 (1.22 – 4.76)** |  |
| Swedish-migrant | - | **2.10 (1.08 – 4.12)** |  |
| Mixed migrant | - | 1.54 (0.66 – 3.61) |  |
| Population density | 1.14 (0.99 – 1.31) | 1.05 (0.94 – 1.18) | 0.4 (1); p=0.55 |
| Neighbourhood own-region migrant density (z-standardised) | 1.09 (0.96 – 1.24) | **1.16 (1.01 – 1.33)** | 0.5 (1); p=0.47 |

^1^Adjusted for age group, sex and other parameters in the table

^2^Likelihood ratio test [LRT] *Χ*^2^ statistic, degrees of freedom [df], and p-value for interaction between migrant status and other variable on odds of compulsory admission

**Supplement Table 5: Distribution of family disposable income by selected region of origin^1^ (n=11,640)**

|  | **Family disposable income quintile (N, %)** | | | | |
| --- | --- | --- | --- | --- | --- |
| **Region** | Low | 2 | 3 | 4 | High |
| Sweden | 374 (5.3) | 1,219 (17.2) | 1,549 (21.8) | 2,049 (28.8) | 1,916 (27.0) |
| Nordic | 45 (19.2) | 52 (22.1) | 54 (23.0) | 41 (17.5) | 43 (18.3) |
| Europe | 180 (24.1) | 140 (18.7) | 171 (22.9) | 171 (22.9) | 85 (11.4) |
| Swedish-Nordic children of migrants | 39 (6.4) | 117 (19.2) | 159 (26.1) | 163 (26.7) | 132 (21.6) |

^1^Χ^2^-test on 12 degrees of freedom, p-value: 474.8, p<0.001
